# Man With Ear Pain

**DOI:** 10.1016/j.acepjo.2026.100440

**Published:** 2026-06-12

**Authors:** Jacob Cabrejas, Wesley Eilbert

**Affiliations:** Department of Emergency Medicine, University of Illinois, College of Medicine Room, Chicago, Illinois, USA

**Keywords:** bullous myringitis, hemorrhagic myringitis, myringitis, otitis media

## 1 Patient Presentation

A 45-year-old man presented to the emergency department complaining of 3 days of left ear pain with a sensation of muffled hearing in the ear. He reported 1 day of cough and nasal congestion prior to the onset of his ear symptoms. On physical examination, he was afebrile with hemorrhagic bullae on his left tympanic membrane (Fig).

**Figure:**
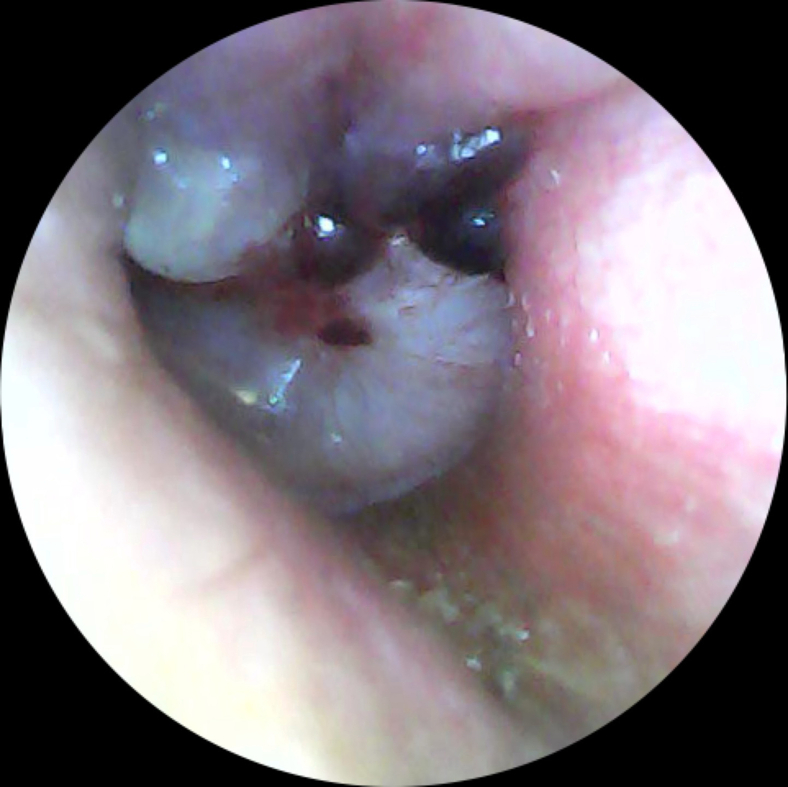
Tympanic membrane with hemorrhagic bullae.

## 2 Diagnosis

### 2.1 Bullous Myringitis

First described in 1891, bullous myringitis (BM) is an acutely painful condition resulting in the formation of bullae on the lateral wall of the tympanic membrane and possibly extending onto the external auditory canal adjacent to the tympanic membrane.^1^^,^^2^ When the bullae are blood filled, the condition is specifically referred to as bullous hemorrhagic myringitis.^3^ BM may be associated with otitis externa, but most cases occur in the presence of otitis media.^4^ BM may occur in all age groups, though it primarily affects children.^2^ BM may occur bilaterally, and as with this patient, patients typically report preceding symptoms of a viral upper respiratory infection.^3^^,^^5^ The ear pain with BM is often of abrupt onset and may radiate to the mastoid process or face.^2^^,^^4^ Fever is present in the majority of cases.^4^ The majority of patients will report hearing loss in the affected ear.^6^

The bacterial and viral pathogens associated with BM are similar to those causing acute otitis media.^7^ Treatment with systemic antibiotics, typically amoxicillin or azithromycin, is indicated.^8^ The pain associated with BM typically resolves in 1 to 2 days, and the hearing loss is transient.^5^^,^^7^

## Funding And Support

By *JACEP Open* policy, all authors are required to disclose any and all commercial, financial, and other relationships in any way related to the subject of this article as per ICMJE conflict of interest guidelines (see www.icmje.org). The authors have stated that no such relationships exist.

## Conflict of Interest

All authors have affirmed they have no conflicts of interest to declare.
